# Training induced fatigability assessed by sEMG in Pre-Olympic ice-skaters

**DOI:** 10.1038/s41598-020-71052-4

**Published:** 2020-08-26

**Authors:** Paweł Pakosz, Mariusz Konieczny

**Affiliations:** Faculty of Physical Education and Physiotherapy, Opole University of Technology, 45-758, Opole, Poland

**Keywords:** Skeletal muscle, Metabolomics

## Abstract

The aim of this study was to investigate the size of the change and asymmetry in fatigability of gluteus maximus muscles during endurance training in short-track. The research has taken into account the position of athletes during skating and the problem of fatigue and pain in these muscles. The research covered involved eight female athletes of the Polish National Team in short track, which had been prepared to the Olympic Games in PyeongChang. The surface electromyography (sEMG) system was used to measure fatigue of right and left gluteus maximus muscles, in the modified Biering–Sorensen test. The test was conducted five times during the training: before training, after warmup, and after each of 3 series of the endurance training. Comparing the mean frequency of the surface electromyography power spectrum of the test, statistically significant reduction of the average frequency value of the right muscle from 55.61 ± 7.08 to 48.64 ± 4.48 Hz and left muscle from 58.78 ± 4.98 to 53.18 ± 4.62 Hz was reported, which prove the muscle fatigue. In subsequent series tests, the sEMG signal frequency of begin decrease more than the end of the each measurement, which determines the fatigue threshold. The size of the d Cohen effect in fatigue drops along with subsequent five tests during the training. Skaters has higher frequency reduction of the right lower limb, which indicates its greater fatigue during skateing. The fatigue and asymmetry in muscle observed in short-track has implications for training and performance.

## Introduction

Speed skating has two disciplines that differ in track length and skating technique. In short track, the distance of one lap is 111 m, which is less than one third of the skating track on a long track (400 m). Such a short distance forces the competitors to go along an oval track with high speed and small turn radiuses. During a race at a distance of 1,000 m the short track competitor, when turning, generates twice as large forces as on a long track^1^. In order to obtain the best possible result in short track, a successful athlete should reduce his fatiguability. In addition, what also matters are the race tactics, psychological and technical aspects, maximum voluntary contraction force, reaction time and the quality of lower limb muscle coordination^2–4^.

General body fatigue can affect psychological and physiological aspects. One of the factors of physiological fatigue is muscle fatigue, which is defined as reduction in muscle capacity to perform work, after prior physical effort^5,6^. Local muscle fatigue is concentrated mainly on a decrease in contraction force, that is inability of the muscle to generate proper capacity^7^. This is an inevitable process, and attempts to reduce it are constantly made. In the literature analysing muscle fatigue, many variants of this process can be found^8^, which also uses surface electromyography and analysis of the sEMG signal frequency of the power spectrum. This method provides useful information on local muscle fatigue^9–12^. When the muscle fatigue increases, the number of active motor units decreases and the speed of conductivity of muscle fibres drops^13^. In addition, motor units are stimulated slower and their work becomes more synchronized^5^. All these changes lead to gradual reduction in the muscular work possibility^14^. Myoelectric manifestations of fatigue are mainly related to the slowing of motor unit action potentials during their travelling along muscle fibers, that is the reduction of their conduction velocity^15^. The second thing is a higher occurrence of simultaneous discharge of action potentials from different motor unit to increase the mechanical output when the whole motor unit pool is recruited^16^. Therefore, to evaluate muscle fatigue, might be estimated during isometric tasks^17,18^, and the sEMG may be here useful.

In fatigue research using sEMG, emphasis was put one the importance of static measurement, that is isometric contraction in isolated muscle position^19^. In such measurement, muscle fatigue is reflected by changes in the amplitude and average frequency of the power spectrum of sEMG signal, and average signal frequencies in all muscle groups tested in this way are decreased. In addition, changes in the amplitude and frequency of the signal are correlated as a result of fatigue, if the signal amplitude increases, then the average signal frequency decreases^10,12,20^. However, there are also reports where physical effort does not affect reduction in the frequency of sEMG signal^21^. In order to perform a correct measurement during isometric contraction, the sEMG signal should be measured between 0.5 and 2 s because only then it gives credible results^22^.

To achieve good results in short track, it is necessary for the athlete to generate high driving force, which translates into the speeds achieved on ice—extensor muscles prove decisive in this task^2,3^. Felser^2^ specifies that the maximum force of lower limb extensors explains 27% of differences in speeds of short track athletes. Neto et al.^23^ demonstrated that training caused reduction in the frequency median for the knee joint extensor muscles in isometric contraction, which proves muscle fatigue. The authors of the following paper analyzed the work of gluteus maximus muscle whose main function is extending the thigh in the hip joint. This selection of the muscle was due to the suggestion of coaches who wanted to learn about the fatigue ranges of the examined muscles.

Despite efforts, the authors of the following paper did not manage to find tests concerning gluteus maximus muscle fatigue in short track athletes using sEMG system. Referring to subject of muscle fatigue, on the other hand it was reported, that athletes going through one-sided curves with high speed, have a tendency to significant asymmetry between local levels of desaturation (reduction in blood saturation with oxygen) in quadriceps femoris muscles of the lower limbs^4^. Therefore, the authors wanted to examine whether there are differences in muscle fatigue between both limbs.

Using the sEMG system to measure the bioelectric activity of muscles in short track athletes during skating, it was confirmed that activation of muscles differs when skating on a straight line and on the curves, as well as in both lower limbs^3^. Activation of the right lower limb is significantly greater during skating at curves as compared to skating on a straight line. This movement pattern was not observed in the left limb.

Better understanding of the nature of work and muscle symmetry in sports, examined by sEMG using the example of short track, may be helpful to coaches, physicians, physical therapists and also athletes. Innovation of the tests presented below consisted also in checking how fatigability is shaped during various training phases. The authors of the work have checked the level of muscle fatigue at the beginning and end of the training and the size of the difference between fatigue of the right and the left gluteus maximus muscle, in spite of a training, based (by definition) on symmetric muscle work.

The following tests, in three aspects, focused on the issue of local muscle fatigue in short track female athletes. One of aspects applies to the fatigue of gluteus maximus muscles that extend femoral joint and substantially affect the strength of lower limb bounce off the ice pane. The second aspect applies to determining the level of asymmetry between the right and the left examined muscle, while the third aspect applies to the size of the fatigue effect.

The aim of this study was to investigate the size of the change and asymmetry in fatigability of gluteus maximus muscles during endurance training in short-track. The first hypothesis of our research is that in the test, mean frequency of power spectrum of the sEMG signal will decrease over 60 s of the modified Biering–Sorensen test and along with the progress of the training. Secondly, owing to the fact that athletes skate always to the left, we expect that the fatigue level will be different for the two lower limbs, and higher in the right limb muscle.

## Results

When checking whether it is possible to demonstrate a significant fatigue level in the Biering–Sorensen test, firstly we measured the mean frequency of the power spectrum of both gluteus maximus muscles (GM), in the first and the last second of the test, in all training series was compared (Table 1). The conducted research shows that in the right muscle this difference amounted on average to 6.97 Hz, and in the left—to 5.60 Hz, and in both cases these are statistically significant changes.

**Table 1:** Values of sEMG signal frequency in gluteus maximus muscles (GM), in the first and the last second of the test.

Variable	Average (Hz)
GM right (1 s)	55.61 ± 7.08
GM right (60 s)	48.64^*^ ± 4.48
GM left (1 s)	58.78 ± 4.98
GM left (60 s)	53.18^*^ ± 4.62

^*^Statistical significance of the changes at the level of *p* < 0.05 as compared to the first second of measurement.

**Figure 1:**
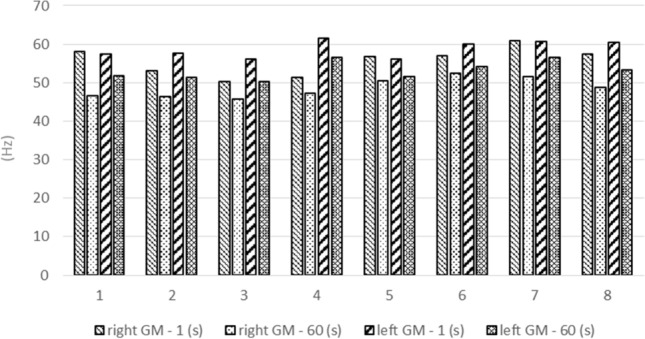
The average value of frequency (Hz) of gluteus maximus muscles of all series, measured for all athletes (1–8).

By analysing the results of athletes individually, it is possible to note clear reductions in the frequency measured in the first and the last second of the test, however, without explicit determination of the left- or the right-sided dominance (Fig. 1).

The results presented in Table 2, show the trend of average frequencies of the first and the last seconds, in subsequent test series. Training causes the results of average frequencies to have a decreasing trend. Considering the first second of the test, the average value of frequency in the right muscles decreased by 9.66 Hz, while in the last second it decreased by 1.2 Hz. In the left limb, similarly, the drops by 3.8 Hz and 1 Hz were observed. Significant changes in frequency between series were demonstrated in 1 (s) of right and left GM tests. In the right GM, significant changes occurred between series 1 and 2 where *p* = 0.05, series 1 and 5, where *p* = 0.02, series 2 and 5, where *p* = 0.04, and series 4 and 5, where *p* = 0.03. In the left GM, significant changes occurred between series 1 and 5, where *p* = 0.04, and series 3 and 5 where *p* = 0.04. Significant changes between series measured in 60 (s), did not occur. A tendency of reduction of the average frequency level in the test, was shown also in an individual test of each of the female athletes. In subsequent test series, of the whole group and of each female athlete separately, a decrease in measurements of average frequencies can be noticed during both the first and the last seconds of the whole one-minute test. Both in the right and in the left gluteus maximus muscle, along with the progressing training, the average frequency measurements drop. It can be observed to a larger extent in the case of the beginning of measurements (average frequencies measured from the first second) than those measured at the end of the measurement (in the last second of the test). The fatigue effect is particularly visible in the first series of the measurements, where the differences between the first and the last second are the largest. In the subsequent measurements, this difference is lower (Table 2).

**Table 2:** Average frequencies in gluteus maximus muscles (GM), in the first and the last second of the test, obtained by athletes in subsequent test series*.*

Variable	1 series (Hz)	2 series (Hz)	3 series (Hz)	4 series (Hz)	5 series (Hz)
GM right (1 s)	60.36	54.86	56.05	56.09	50.70
GM right (60 s)	48.61^*^	50.45^*^	47.54^*^	49.17^*^	47.42^*^
GM left (1 s)	59.80	58.97	62.28	56.86	55.97
GM left (60 s)	52.62^*^	54.55^*^	54.74^*^	52.36^*^	51.62^*^

^*^Statistical significance of the changes at the level of *p* < 0.05, as compared to the first second of measurement.

Measuring the size of the d Cohen effect, assessing the fatigue between the 1st and 60th s. of the test, it is possible to note a strong effect (value > 0.5) for each series (Table 3). The size of the effect drops along with subsequent training series. In the right gluteus maximus muscle, the strongest effect of fatigue is visible in the first series, and the lowest-in the last series. In the left muscle, there is a higher variability of results.

**Table 3:** The size of the d Cohen effect of gluteus maximus (GM) muscles fatigue, obtained by athletes in subsequent test series.

Variable	1 series	2 series	3 series	4 series	5 series
GM right (d Cohen effect)	1.73	1.26	1.16	1.10	0.90
GM left (d Cohen effect)	1.79	1.14	1.19	1.16	0.90

## Discussion

The aim of this study was to investigate the size of the change in fatigability and asymmetry of gluteus maximus muscles during difficult endurance training. Conducting such tests is one of the greatest challenges for science in sports. Achievement of the best performance is strictly related to optimization of the training process, and this thesis, concerning the fatigue of gluteus maximus muscles in short track athletes is to contribute to this issue. Many aspects concerning fatigue in short track, have not been tested yet, because it is not a very broadly examined sports discipline, and the training process requires enhancements. In order to obtain the best training model, it is necessary to have accurate knowledge of sports requirements specific for this discipline, including individual improvement of each athlete^2^. Physiological symptoms of muscle fatigue strongly affect and decreases their frequency measured by sEMG^24^, however, in the opinion of Peach et al.^25^, such fatigue measurements cannot be relied upon. In order to avoid an error, we repeated our test in five series. In order to test muscle fatigue using the sEMG system, we paid attention first to the claim by Petrofsky^19^ concerning a test during isometric contraction in isolated muscle position conducted in the Biering–Sorensen test. Our tests revealed a decrease in mean frequency of the power spectrum of the sEMG signal during the test, as well as in the course of consecutive stages of the training, which confirmed Petrofsky research.

The results of the conducted tests also demonstrated usefulness of sEMG in determination of muscle fatigue, which is consistent with the tests by other authors^9,11,26,27^. In addition, we demonstrated that changes in average frequencies measured in the first second, at the beginning and at the end of the test, vary statistically in a substantial way. As a result, the Biering–Sorensen test may be regarded a reasonable tool for testing muscle fatigue, which is suggested by, among others, Champagne et al.^28^, Howard et al.^29^, Larivière et al.^30^. In the studies presented, along with subsequent series, the average frequencies of the first measured second have a decreasing trend to a greater extent than those measured at the end of the measurement, which additionally are similar in each series. On this basis, it can be concluded that in the last second of the measurement, the frequency values reach the fatigue threshold which the organism cannot exceed. Therefore, it is possible to develop the reports of authors Al-Mulla et al.^8^ who believe that there is an individual variability of muscle features and it is not possible to simply determine the load and time function precisely specifying the muscle fatigue threshold.

Felser et al.^31^, showed the differences in the asymmetry of bioelectrical muscle activity during skating in different sections of the track on ice. The authors showed the occurrence of greater bioelectrical activity in the right leg during cornering on the track. Hesford et al.^32^ also observed this phenomenon when exploring oxygenation of leg muscles, during cornering on the track. De Boer et al.^33^. they noticed, that comparing the skating on a straight line and on corners, there were much larger changes in joint movement in the right lower limb. In the right limb there were still exesting changes, and in the right ankle the plantar flexion phase of the foot was extended, which is the main mechanism that generates repulsive force from ice^34^. Noticing these differences between the sides, it can be stated that fatigue of the muscles of right and left lower limb will differ. Training result, was higher changes in frequency in the right muscle, comparing the measurements from the beginning and the end of the training. It would be a confirmation of a generally known trend in short track, involving higher load, and thereby higher fatigue of the right lower limb during skating. This is an effect of its higher load on curves^31^. Our test took place during specialized training, where athletes were skating along a track straining the organism in the aspect of endurance and speed at the same time. The speeds of the female athletes were high, and so were the strains of lower limbs. In the short track athletes, the right lower limb muscles work to a greater extent when skating on the curves on one track lap^4,31^.

More studies are needed to understand the implications of this phenomenon. In future research, it is worth investigating whether alterations in technique and training methods can improve performance assisted by sEMG. Based on the conducted research, we suggest to extend the analysis also to other muscles in the lower limbs, which would give more precise information about muscle fatigue. Using the EMG method for assessment of the gluteus maximus muscle fatigue in short track athletes, high usefulness has been demonstrated. Studies have shown a general trend in asymmetry of gluteal muscle fatigue in short-track. Coaches should also take into account the fact of increased fatigue in the right muscle when planning the training process. The authors suggest increasing pressure on the post-training process of the right limb. In addition both, coaches and the athletes themselves should be aware of what their skating technique looks like and how and when individual parts of their body work. Thanks to this, better results at lower energy costs, as well as prevent future injuries, can be achieve.

## Conclusions

The muscle signal frequency measured by sEMG decreases in one-minute Biering–Sorensen test, which prove the fatigue of muscles. In subsequent series tests, the frequency of 1 s of the measurement has a higher decreasing trend than the measurement of the last second of the measurement, which determines the fatigue threshold. Reductions in the frequency measured in the first and the last second of the test, was higher of the right lower limb. The size of the d Cohen effect in fatigue decreases with subsequent training series.

## Material and methods

### Participants

Tests were performed on eight female athletes of the senior Polish national team in short track who had no medical conditions within the measured muscles and joints that may have an impact on the course and result of the test. It is a group of athletes aged 18.7 ± 2.9 years, with body height of 162 ± 2.4 cm, and body weight of 57.2 ± 5.9 kg. The research was conducted directly during the start-up period the Olympic Games in PyeongChang. During this period, the Polish Women's National Short Track Team recorded the greatest development in the history of the Polish short track. Poland qualified for the Olympics Games and was the team, which obtained the highest progress from year to year from all other countries.

The research was conducted after a weekend break in training to avoid the effect of effort-related fatigue accumulation. The participants were informed about the purpose and the course of tests, and signed a permission to participate in the tests, approved by Bioethical Commission of the Chamber of Physicians in Opole No. 237, in accordance with the guidelines specified in the Declaration of Helsinki on human experimentation. The respondents were asked to refrain from intensive physical exercises in the period of 1 day preceding the test, and not to consume meals and beverages containing caffeine at the interval of 3 h before the test. The research was conducted in the presence of a coach. The authors confirm that all research was performed in accordance with relevant guidelines, and obtained the informed consent of all participants.

It is extremely important for the tests to be as similar as possible to the realities of sports competition. In one of the tests, it was demonstrated that the size of deoxidation of muscles when skating on ice, at the same time in forced lowed body position, was much higher than that observed during the tests on a treadmill^35^. This suggests that the tests should be conducted on ice to obtain the most realistic reflection and understanding of the conditions prevailing in speed skating. In order to come as close as possible to the ideal conditions, tests were conducted at a skating rink and athletes were tested directly after finishing a series of exercises. The tests took place with full uniform and electrodes and sEMG sensors were constantly fastened to the athletes throughout the whole training.

### Procedure

#### Biering–Sorensen test

Frequency of the power spectrum of the sEMG signal (reflecting the muscle fatigue level) was examined by means of a sEMG signal in the body position used in the Biering–Sorensen test in isometric contraction conditions. The conducted tests involved the use of repeatedly mentioned in the literature Biering–Sorensen test in which the anti-gravitational body position provides isometric and symmetrical tension of lower limb and spinal muscles. The subject was lying on a examining table. Test was performed in the prone position, with the iliac crests aligned with the table edge and the lower limbs fixed by straps at the ankles and below the knees. During each test, the subjects were instructed to keep their body (head, arms and trunk) unsupported, horizontal to the ground, as long as they could, with their arms crossed at the chest. Verbalized encouragement was provided throughout the test. The subjects were also instructed to maintain the lumbar lordosis position as stable as possible. Duration of the test was limited to 60 s, as in the case of Katakura et al.^26^. The test duration was shortened because execution of the trial until the so-called “refusal” could cause excessive strain for the competitors and failure to achieve the training goals. The fatigue tests in isometric contraction over 60 s were also conducted by other authors, modifying the test to their own needs in terms of positions and duration^28–30^. Analyses took account of changes between the first and the last second of the registered sEMG record. They mainly determined if the EMG variable decreased from the first to last second of Sorensen test. The test protocol for testing fatigue of gluteus maximus muscle during training, contained 5 series for each of the examined athletes. All study participants successfully completed each series of the 60 s test.

#### Training process

**Figure 2:**
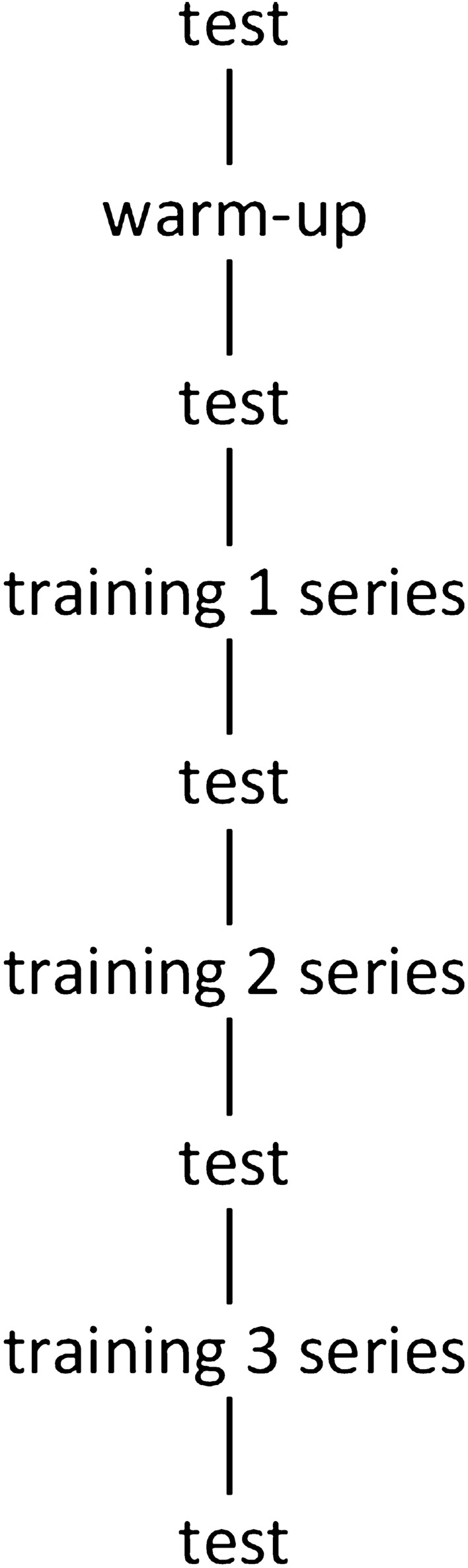
Time scheme of testing.

The first series of tests were held before the training. The next series followed the 15 min general warm-up on a track. The last three tests were conducted subsequently after each of 3 series of the endurance training, conducted on ice with full uniform (Fig. 2).

Each of the three series of endurance training consisted of nine sub-series. The one-minute sub-series consisted of skating on ice with submaximal load and a one-minute break between. The breaks between series in which the test was performed took 8 min. After 3 series of endurance training, there was 10 min cool down. During the training, the trainer split the study group so that during training the athletes reach the submaximal load (90–95% heart rate) determined by the trainer in each series.

#### Research process

The competitors started each examination lying on a medical examination couch. Their lower limbs were located on the surface of the couch, and the anterior superior iliac spines line, hung down beyond the edge of the couch. During the test, the legs were stabilized, and the torso dangled freely outside the couch. At the start signal, the athlete raised his torso to the level of the lower limbs, and was supposed to hold such posture for 60 s of the test. Examination couch was placed on the skating rink, and the tests after each of 3 training series, took place immediately after their completion.

#### Data collection

The test of bio-electric activity of the right and left gluteus maximus muscle, was conducted by means of electromyograph TeleMyo DTS (NORAXON). Before the test, the place for sticking electrodes was shaved and cleaned using alcohol-soaked cotton to minimize skin impedance. Bipolar electrodes (Ag/AgCl) had a pre-gelled diameter of 10 mm and the inter-electrode distance was 2 cm. Surface electrodes were placed on the muscle venter between the movement point and the tendon attachment, along the longitudinal middle line of the muscle, according to SENIAM methodology^28,36^.

The NORAXON DTS system had the following technical specification: basic noise of the device, below 1 µV RMS, input impedance above 100 Momh, CMR (common signal rejection factor) greater than 100 dB, sampling frequency of 1,500 Hz, reinforcement: 500. The raw EMG signals were processed into a root mean square (RMS) with a window of 50 ms. A band pass filter of 20–450 Hz was used together with notch filters at 60 Hz. Processing the signal and EMG analysis were performed using NORAXON MyoResearch-XP 1.07 Master Editionx software.

### Statistical analysis

The statistical analysis was conducted using STATISTICA 12.0 Because the distribution wasn't normal, so to determine the significance of differences, the Wilcoxon test was used, and the differences were recognized as statistically significant, if the similarity of the examined variables was lower than the assumed significance level *p* ≤ 0.05. In order to determine the strength of association between variables the size of the d Cohen effect was calculated. Cohen's d was determined by calculating the mean difference between your two groups, and then dividing the result by the pooled standard deviation. Effect size “small, d = 0.2,” “medium, d = 0.5,” and “large, d = 0.8”.

### Ethical approval

All procedures performed in studies involving human participants were in accordance with the ethical standards of the institutional and/or national research committee and with the 1964 Helsinki declaration and its later amendments or comparable ethical standards.
